# Growth and structural characterisation of Sr-doped Bi_2_Se_3_ thin films

**DOI:** 10.1038/s41598-018-20615-7

**Published:** 2018-02-01

**Authors:** Meng Wang, Dejiong Zhang, Wenxiang Jiang, Zhuojun Li, Chaoqun Han, Jinfeng Jia, Jixue Li, Shan Qiao, Dong Qian, He Tian, Bo Gao

**Affiliations:** 1 CAS Center for Excellence in Superconducting Electronics (CENSE), Shanghai, 200050 China; 2 Center of Electron Microscopy and State Key Laboratory of Silicon Materials, School of Materials Science and Engineering, Zhejiang University, Hangzhou, 310027 China; 3 Key Laboratory of Artificial Structures and Quantum Control (Ministry of Education), School of Physics and Astronomy, Shanghai Jiao Tong University, Shanghai, 200240 China; 4 State Key Laboratory of Functional Materials for Informatics, Shanghai Institute of Microsystem and Information Technology, Chinese Academy of Sciences, 865 Changning Road, Shanghai, 200050 China; 5 Collaborative Innovation Center of Advanced Microstructures, Nanjing, 210093 China; 6 University of Chinese Academy of Sciences, Beijing, 100049 China

## Abstract

We grew Sr-doped Bi_2_Se_3_ thin films using molecular beam epitaxy, and their high quality was verified using transmission electron microscopy. The thin films exhibited weak antilocalisation behaviours in magneto-resistance measurements, a typical transport signature of topological insulators, but were not superconducting. In addition, the carrier densities of the non-superconducting thin-film samples were similar to those of their superconducting bulk counterparts. Atom-by-atom energy-dispersive X-ray mapping also revealed similar Sr doping structures in the bulk and thin-film samples. Because no qualitative distinction between non-superconducting thin-film and superconducting bulk samples had been found, we turned to a quantitative statistical analysis, which uncovered a key structural difference between the bulk and thin-film samples. The separation between Bi layers in the same quintuple layer was compressed whereas that between the closest Bi layers in two neighbouring quintuple layers was expanded in the thin-film samples compared with the separations in pristine bulk Bi_2_Se_3_. In marked contrast, the corresponding changes in the bulk doped samples showed opposite trends. These differences may provide insight into the absence of superconductivity in doped topological insulator thin films.

## Introduction

Topological insulators (TIs) and superconductors (TScs) are active research fields in condensed matter physics^1^. The ability of TScs to host gapless Majorana-type collective excitations^2,3^, which can serve as building blocks for fault-tolerant topological quantum computing^4–6^, has sparked substantial research interest. One possible route to obtain TScs is to dope TIs. To date, superconductivity has been successfully induced in doped TIs such as Cu-, Sr-, and Nb-doped Bi_2_Se_3_^7–9^. A few signs of topological superconductivity have been observed in these materials. For example, point contact spectroscopy measurements have revealed a zero-bias conductance peak (ZBCP) in Cu-doped Bi_2_Se_3_, suggesting a possible mid-gap state that may be related to the Majorana zero mode^10^. Signs of odd-parity superconductivity have also been inferred from various measurements including nuclear magnetic resonance experiments and angle-dependent specific heat measurements^11,12^. However, contradictory results have also been reported. A scanning tunnelling spectroscopy study of Cu-doped Bi_2_Se_3_ did not reproduce the finding of ZBCP^13^. The key problem is the relatively low superconducting volume fraction (~40%)^7,14–17^ and large superconducting inhomogeneity in single crystals^13,16^. To search for smoking-gun type evidence of Majorana zero modes, a number of detection schemas have been proposed, many of which require the preparation of superconducting films from doped TIs^18–22^. Unfortunately, attempts to grow superconducting doped TI films have not yet been successful^23–25^. The superconductivity in Cu-doped Bi_2_Se_3_ was initially believed to originate from the intercalation of Cu dopant atoms into van der Waals (vdW) gaps. However, although Cu intercalation was successfully realised in Bi_2_Se_3_ thin films grown by molecular beam epitaxy (MBE), the Cu-doped Bi_2_Se_3_ films were not superconducting^23^. Here, we report our attempt to grow Sr-doped Bi_2_Se_3_ thin films using MBE. High-resolution high-angle annular dark-field scanning transmission electron microscopy (HAADF-STEM) examination verified the high quality of the films. In addition, magneto-resistance measurements revealed a weak antilocalisation (WAL) behaviour, which is a typical transport signature of TIs^26–30^, indicating the well-preserved topological surface state. Similar to Cu-doped Bi_2_Se_3_ thin films, the Sr-doped Bi_2_Se_3_ thin films were not superconducting, although the carrier densities of the films were similar to those of superconducting bulk Sr_x_Bi_2_Se_3_ samples^8,31^. To explore the differences between the non-superconducting thin films and superconducting bulk samples, we performed atom-by-atom energy-dispersive X-ray spectroscopy (EDX) mapping. Similar Sr doping structures were observed in both types of samples. The only difference was the opposite trend of expansion/compression of the separation between Bi layers in the Bi_2_Se_3_ lattice for the bulk and thin-film doped samples (compared with that in pristine Bi_2_Se_3_), which suggests that the emergence of superconductivity in doped Bi_2_Se_3_ is possibly related with doping-induced lattice structural change.

## Results

### Structural and electrical characterisation of Sr-doped Bi_2_Se_3_ thin films

**Figure 1:**
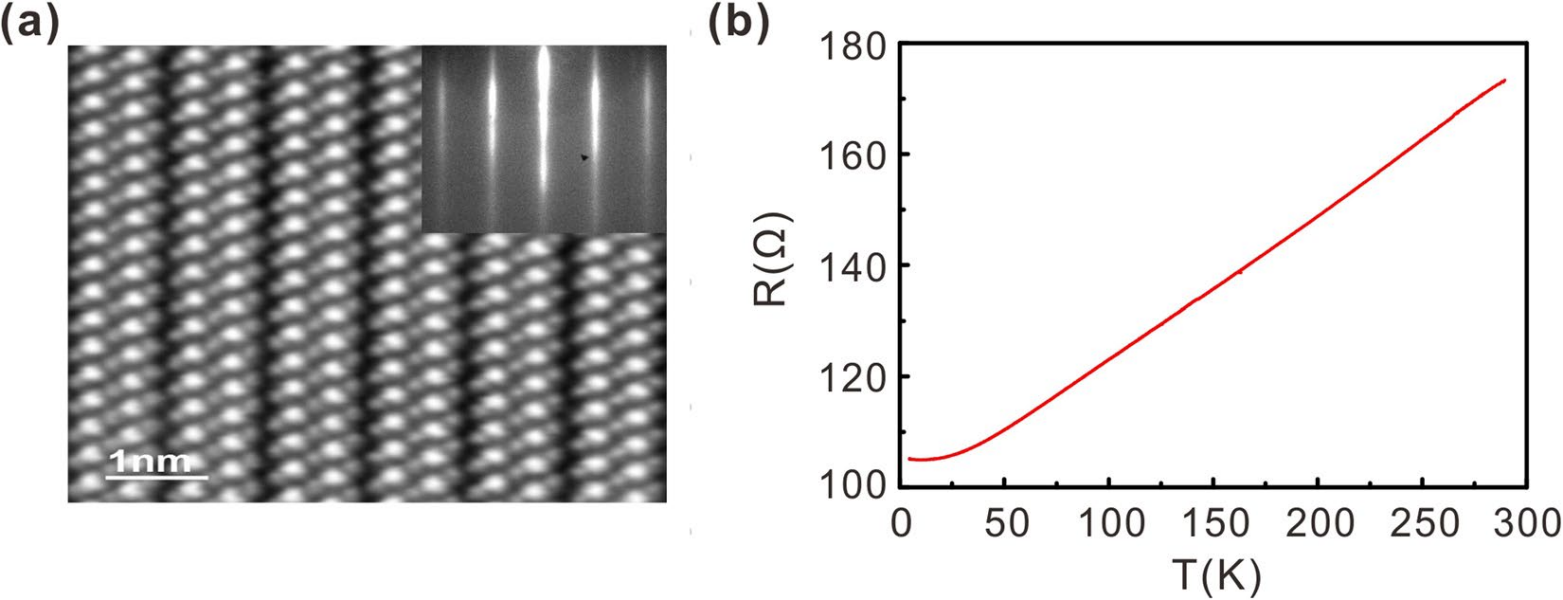
Structural and electrical characterisation of Sr-doped Bi_2_Se_3_ thin films. (**a**) Cross-sectional HRTEM image of a film clearly showing the quintuple-layer structure. Inset: Resolved RHEED pattern of a Sr-doped Bi_2_Se_3_ thin film. **(b)** Typical temperature dependence of the resistance of Sr-doped Bi_2_Se_3_ thin films.

Sr-doped Bi_2_Se_3_ thin films were grown using MBE, and HAADF-STEM was used to inspect the film quality. Figure 1a presents a HAADF-STEM image of a thin-film sample, clearly revealing the typical quintuple-layer structure of Bi_2_Se_3_. The bright columns correspond to Bi atoms with high atomic number. The Sr dopant atoms are difficult to detect in the HAADF-STEM image because of the low Sr concentration of the sample and the low atomic number of Sr. The inset of Fig. 1a presents a reflection high-energy electron diffraction (RHEED) pattern of an approximately 50-nm-thick film, which confirms the high film quality. To determine whether the thin-film sample was superconducting similar to bulk Sr-doped Bi_2_Se_3_, we performed resistance versus temperature (R–T) measurements, as shown in Fig. 1b. The R–T measurements do not reveal any sign of superconducting behaviour.

**Figure 2:**
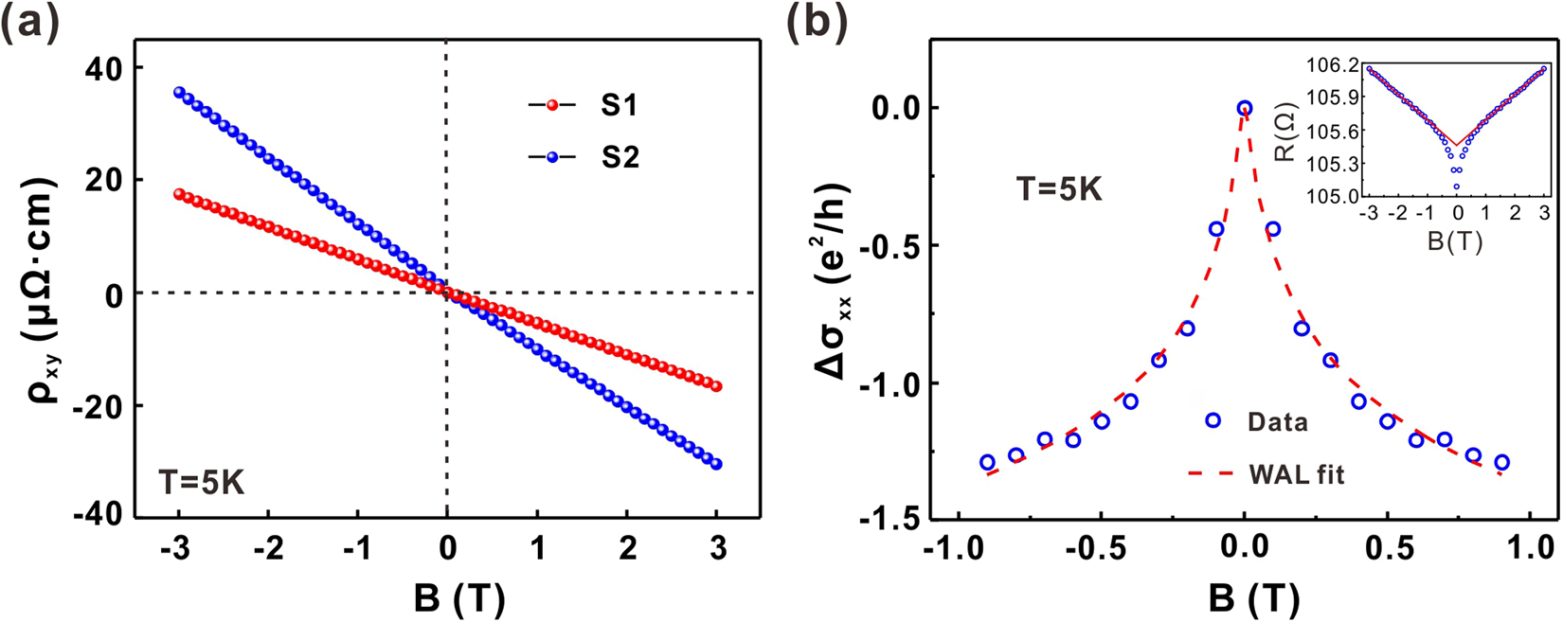
Hall measurements and WAL behaviour of Sr-doped Bi_2_Se_3_ thin films. (**a**) Hall resistivity versus magnetic field of two Sr-doped Bi_2_Se_3_ thin films measured at 5 K. (**b**) WAL behaviour of the thin films at 5 K. The red dashed line in the low-magnetic-field region is a WAL fit to the magneto-conductivity, which was subtracted by the conductivity background deduced from the extrapolating linear magneto-resistance towards zero magnetic field. Inset: The red solid line is a linear fit to the magneto-resistance in the region of magnetic field $|B|\ge 1{\rm{T}}$, which contributes the conductivity background from the bulk.

The carrier densities in the Sr-doped thin-film samples were determined using Hall measurements. Figure 2a presents the Hall resistivity curves of two thin-film samples. The carrier densities deduced from the linear fitting of the curves were 1.11 × 10^20^ cm^−3^ and 5.67 × 10^19^ cm^−3^ at 5 K, respectively. These numbers are close to the typical carrier density of bulk superconducting Sr-doped Bi_2_Se_3_ samples^8,31^. In addition, a prominent cusp of magneto-conductivity was observed near zero magnetic field at 5 K, as shown in Fig. 2b. The cusp can be attributed to WAL behaviour, which is a typical transport signature of two-dimensional topological surface states and has been observed in many transport measurements of TI thin films^26–30^. Usually the WAL behaviour can be analysed using the Hikami–Larkin–Nagaoka (HLN) quantum interference model^32^. We also noticed that Adroguer *et al*. has proposed a new model to describe the quantum correction to conductivity, which accounts for the Dirac nature of the surface state while HLN model considers a quasi-two-dimensional electron gas with parabolic electron dispersion^33,34^. Because the carrier density of thin-film samples is in the order of 10^19^~10^20^ cm^−3^, the Fermi level (approximately 320~400 meV above the Dirac Point) crosses with the bulk conduction band according to our previous angle-resolved photoemission spectroscopy (ARPES) measurement results on bulk Sr-doped Bi_2_Se_3_ samples with similar carrier density^35^. In this condition, the HLN model can still be applied to our samples:

1 $${\rm{\Delta }}{\sigma }_{xx}\,(B)\,=-\alpha \cdot \frac{{e}^{2}}{\pi h}[\psi (\frac{1}{2}+\frac{{B}_{\varphi }}{B})-\,\mathrm{ln}(\frac{{B}_{\varphi }}{B})]$$

where ${\rm{\Delta }}{\sigma }_{xx}\,(B)={\sigma }_{xx}\,(B)-{\sigma }_{xx}\,(0)$ represents the variation of two-dimensional magneto-conductivity, $\alpha$ is the WAL coefficient, $\psi$ is the digamma function, and ${B}_{\varphi }=\frac{\hslash }{4e{l}_{\varphi }^{2}}$ is the effective magnetic field characterised by the dephasing length ${l}_{\varphi }$. The $\alpha$ value can be 0.5 or 1 depending on the number of topologically protected transport channels. In addition to the cusp near zero magnetic field, the magneto-resistance measurements also revealed a linear magnetic field dependence, as fitted in the inset of Fig. 2b. This linear magneto-resistance behaviour has been observed in many transport measurements of TIs and has been attributed to the scattering of Dirac electrons in the surface transport channel and the impurity scattering of the electrons in the bulk transport channel^27,28,36–38^. To achieve a better fit for the WAL behaviour, we subtracted the conductivity deduced from the linear magneto-resistance background from the raw data by extrapolating the linear magneto-resistance towards zero magnetic field^27^. The fitting yielded values of $\alpha =1.28$ and ${l}_{\varphi }=183\,{\rm{nm}}$, which agrees well with previous measurements for Bi_2_Se_3_^39–41^.

**Figure 3:**
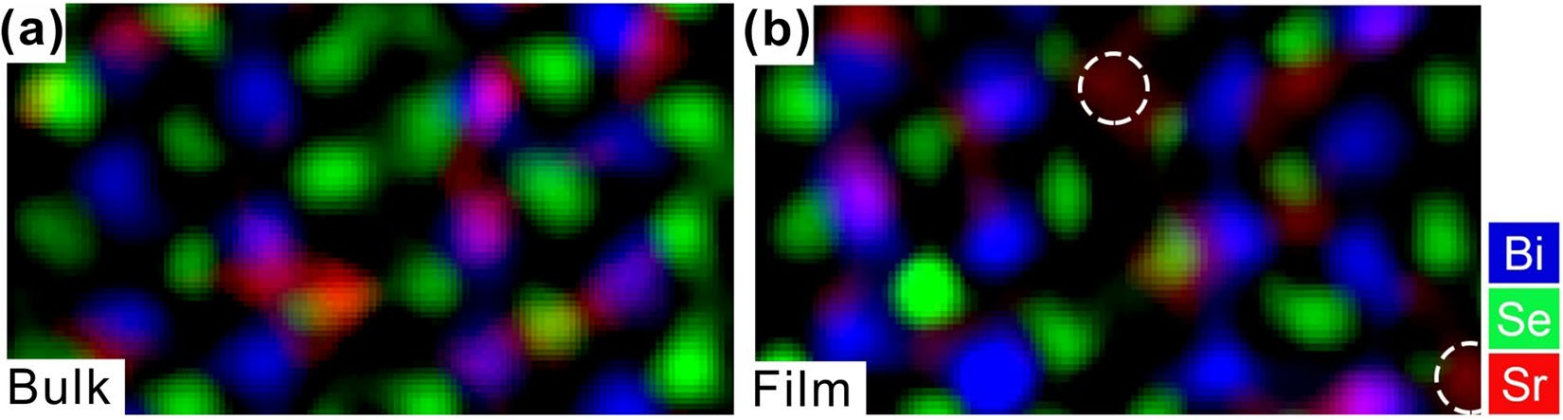
EDX mappings of bulk and thin-film samples. (**a**) EDX mapping for superconducting Sr_0.05_Bi_2_Se_3_ bulk sample and **(b)** Non-superconducting thin-film Sr_0.13_Bi_2_Se_3_ sample. The white dashed circles denote the Sr dopant atoms located in vdW gaps.

To explore the differences between the superconducting bulk and non-superconducting thin-film samples, we performed atom-by-atom EDX mapping for both types of samples. Figure 3a and b present the EDX mappings for the superconducting Sr_0.05_Bi_2_Se_3_ bulk (Sample A) and non-superconducting thin film Sr_0.13_Bi_2_Se_3_ (Sample B), respectively. No clear differences were observed. Bi(Sr) substitutional and interstitial doping were detected inside the quintuple layers in both types of samples. The slight difference is that Sr dopant atoms intercalated in vdW gaps were frequently visible in the thin-film samples, whereas their presence in the bulk samples was relatively rare. This difference is mainly due to the technical difficulty in imaging the dopant atoms in vdW gaps. Because the dopant atoms are highly mobile in vdW gaps, the bulk samples with low Sr concentration (Sr_0.05_Bi_2_Se_3_) can hardly generate signals in EDX mapping as strong as the thin-film samples with higher Sr concentration (Sr_0.13_Bi_2_Se_3_) can.

### Statistical method to determine the separations between adjacent Bi layers

**Figure 4:**
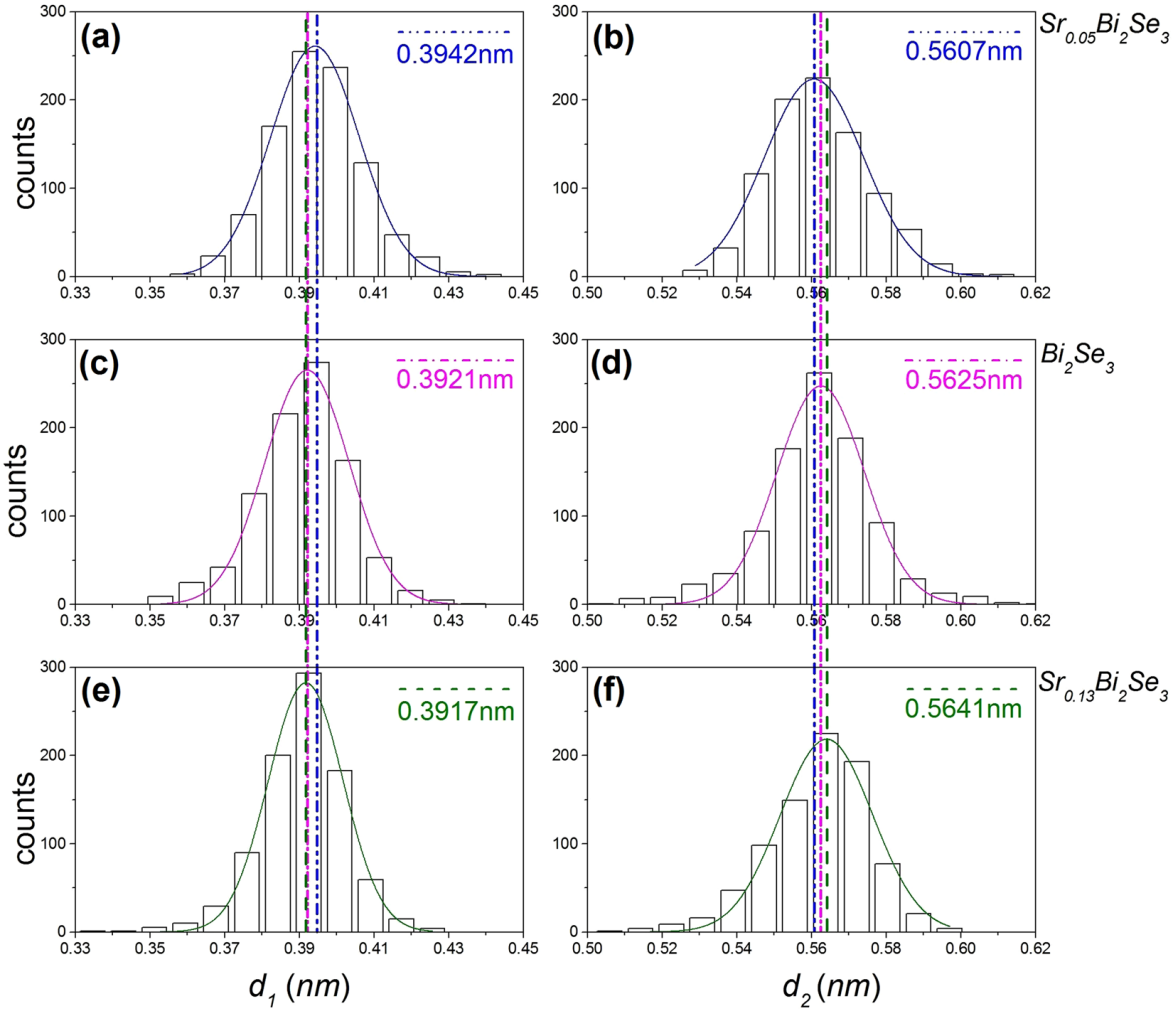
Statistics of the separation between Bi layers in the same quintuple layer (*d*_1_) and between the closest Bi layers in two neighbouring quintuple layers (*d*_2_). (**a**,**b**) Sr_0.05_Bi_2_Se_3_ bulk sample; (**c**,**d**) pristine Bi_2_Se_3_ bulk sample; and (**e**,**f**) Sr_0.13_Bi_2_Se_3_ thin-film sample. *d*_2_ of the Sr_0.13_Bi_2_Se_3_ thin-film sample was clearly expanded by 3.4 pm compared with that of the Sr_0.05_Bi_2_Se_3_ bulk sample, whereas *d*_1_ was compressed by 2.5 pm.

**Figure 5:**
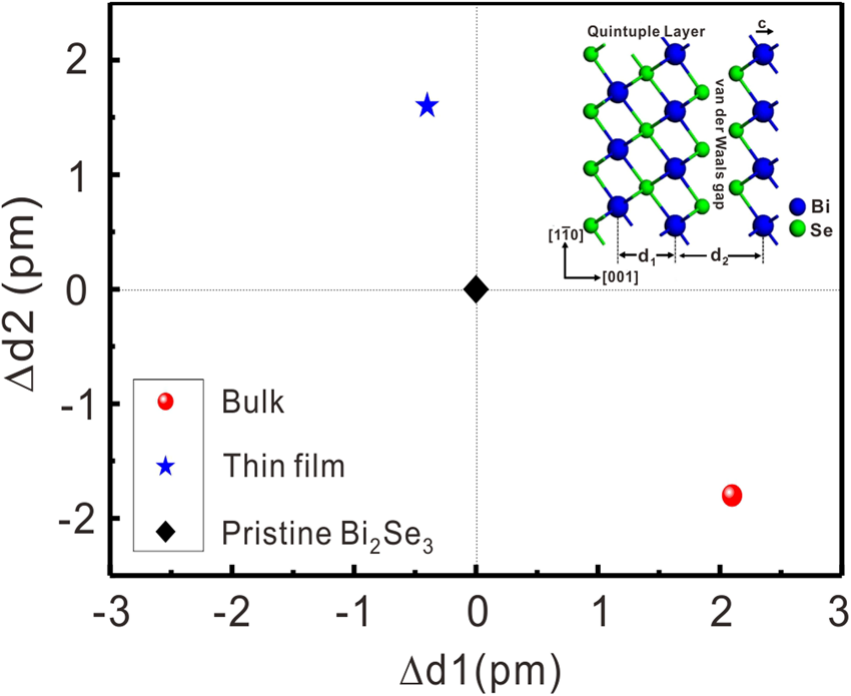
Lattice deformation deduced from STEM measurements. Variations of d1 and d2 in bulk and thin-film samples, with pristine Bi_2_Se_3_ used as a reference. d1 is the spacing between two Bi layers in the same quintuple layer, and d2 is the spacing between the closest Bi layers in two neighbouring quintuple layers. Inset: Schematic illustration of d1 and d2 in Bi_2_Se_3_ lattice.

To further explore the differences between the bulk and thin-film samples, we quantitatively evaluated the variation of the Bi_2_Se_3_ lattice structure along the c-axis. In addition to samples A and B, a pristine Bi_2_Se_3_ bulk sample (sample C) was prepared for comparison. We performed numerous measurements of the separation between two Bi layers inside the same quintuple layer (d1) and between the closest Bi layers in two neighbouring quintuple layers (d2) for these samples. To reduce any systematic errors, all the high-resolution HAADF-STEM images were obtained under the same experimental settings. The original images were fast Fourier filtered to reduce the noise, which also enhanced the image contrast. Because Bi atoms are much heavier than Se atoms, their intensity was higher. Thus, by setting an appropriate image intensity threshold, all the Bi atoms could be identified along with their atomic locations. The actual position of each individual atom was determined from the two-dimensional Gaussian peak fitting of the image intensity. Using the method described above, the separation between adjacent Bi atoms was measured with picometre accuracy. Figure 4 presents a histogram of the separations d1 and d2 for samples A, B, and C. Using pristine Bi_2_Se_3_ as a reference, the Gaussian fitting of the histograms indicated that d1 in Sr_0.05_Bi_2_Se_3_ bulk was expanded by 2.1 pm, whereas d2 was compressed by 1.8 pm; in contrast, in the thin-film sample, d1 was compressed by 0.4 pm and d2 was expanded by 1.6 pm. Therefore, the structural changes in the bulk and thin-film Sr-doped Bi_2_Se_3_ samples showed opposite trends of compression/expansion, as illustrated in Fig. 5.

## Discussion

Why superconductivity is absent in doped TIs thin films has been elusive for a long time. The answer to this question is not only necessary to understand the origin of the superconductivity in doped TIs, but will also contribute to the successful growth of superconducting doped TI thin films, which are important for the study of exotic Majorana quasiparticles and the related quantum transport phenomena.

Initially, it was thought that dopants such as Cu atoms intercalated into vdW gaps acted as electron donors and that the superconductivity was generated merely through electron doping. Later experiments performed by Shirasawa *et al*., however, did not support this speculation^23^. Although Cu intercalation was confirmed in their thin Bi_2_Se_3_ film grown by MBE, the expected superconductivity did not appear. The authors concluded that the electron doping itself could not guarantee the emergence of superconductivity and that other effects such as inhomogeneity may also be vital for superconductivity. There were some suspicions, for example in Cu doped Bi_2_Se_3_, that copper atoms might leak out to the surface, especially in thin-film and nano-flake samples, and the leakage of copper atoms would destroy the superconductivity. For example, Ribak *et al*. occasionally observed an extremely large band gap in Cu-doped Bi_2_Se_3_ superconducting bulk samples using angle-resolved photoemission spectroscopy^42^. They found through calculations that the application of a uniaxial internal stress along the c-axis could explain the increase of the band gap. They thought that the release of the stress in the layers close to the surface might explain why it was rare to observe such a large band gap in previous ARPES measurements. They claimed that the absence of superconductivity in exfoliated Bi_2_Se_3_ was also related with the release of the internal stress^42^.

In our experiments, we found that Sr doped Bi_2_Se_3_ thin-film samples resembled their bulk counterparts in many aspects. TEM inspection revealed good crystallinity of the thin-film samples, and magneto-resistance measurements confirmed the existence of a topological surface state. In addition, the carrier densities of the thin-film samples were similar to those of the bulk Sr-doped Bi_2_Se_3_ samples. Even the EDX mappings did not reveal any clear differences in the Sr doping structures between the bulk and thin-film samples. Since our films are quite thick, it is unlikely that the absence of superconductivity is due to the direct tunnelling between the top and bottom surfaces^43,44^. Because the superconducting properties of doped Bi_2_Se_3_ should be mainly affected by its doping structure, the emergence of superconductivity must be highly corelated with the subtle variations of Bi_2_Se_3_ lattice structure (One can also argue that the superconductivity is caused by an unknown impurity. But to date, no such impurity has been identified yet).

As the technique used in the growth of thin-film and bulk samples is very different, and the actual Sr concentration depends on many experimental details such as the choice of substrate, the environmental temperature during sample growth and the nominal Sr doping level, it will be very hard to get identical actual Sr concentration in thin-film and bulk samples to make a more robust comparison. We think that the best strategy is to turn a non-superconducting film into a superconducting one, then one can make a comparison of lattice constants between a superconducting film and a non-superconducting one grown under similar conditions. We noticed a recent work from Mlack *et al*. who used voltage pulse/thermal annealing to treat Bi_2_Se_3_ nano-flakes (approximately 100-nm-thick) capped with Pd electrodes^45,46^. Although the superconducting behavior observed in the annealed flake was not very clean, this work shows promise that superconducting doped Bi_2_Se_3_ thin film is possible to get.

In summary, the emergence of superconductivity in doped Bi_2_Se_3_ should be highly corelated with the doping structure. Because no qualitative distinction between superconducting bulk and non-superconducting thin-film samples has been found, one should turn to quantitative structural analysis to understand the emergence of superconductivity in doped Bi_2_Se_3_. Future work with better design of experiments is needed to reveal the relation between superconductivity and doping induced lattice structure changes.

## Methods

### Material Synthesis

Sr_0.05_Bi_2_Se_3_ superconducting bulk materials were synthesised by melting a mixture of high-purity Bi, Se, and Sr with a nominal atomic ratio of 2:3:0.05. The mixture was prepared in a nitrogen glove box and then sealed in a quartz ampoule. The ampoule was heated at 850 °C for 24 h and then cooled to 620 °C at a rate of 3 °C/h. The samples were then quenched in ice water. The Sr-doped Bi_2_Se_3_ thin-film samples were grown by MBE on insulating SrTiO_3_(111) substrates. The substrates were heated at approximately 240 °C during film growth. Bi and Se were co-deposited onto the substrate with a flux ratio of ~20:1.

### Sample Characterisation

The electrical measurements were performed in a helium-4 cryostat and in a dilution fridge using DC and the lock-in technique.

### HRTEM Examination

Cross-sectional samples were prepared for HRTEM examination using a dual-beam microscope (FIB, Quanta 3D, FEG, FEI) with Ga ion milling and a precision ion-polishing system (Gatan 691) with Ar ion milling. The structural defects of the samples were examined with an FEI TITAN Cs-corrected ChemiSTEM operated at an acceleration voltage of 200 kV to avoid knock-on damage. HAADF-STEM analysis was performed using a spherical aberration probe-corrector to achieve a spatial resolution of up to 0.08 nm. The ChemiSTEM EDX provides outstanding sensitivity for determining elements at atomic resolution. To minimize the uncertainty of our measurements, sample drafting was well controlled in the experiments. Any data with an average drafting large than 0.1 pm/unit cell was excluded. To minimize the artificiality caused by different experimental conditions, the three samples reported in the text were measured successively in short delay and under nearly the same conditions, including the way of sample preparation, thickness of sample, magnification, camera length, pixel size, probe size, and so on.
